# Mental Foramen Position, Shape, Continuity, and Symmetry Among Malocclusion Patients: A Radiographic Study

**DOI:** 10.7759/cureus.51056

**Published:** 2023-12-25

**Authors:** Ahmed Abdullah Bahamid, Sarah Yousef Alsaif, Amal Samir Mohamed Almansouri, Shoug Mefawez Alshammari, Faten Abdullah Alshahrani, Hibah Ali Alhusayni

**Affiliations:** 1 Department of Preventive Dentistry, College of Medicine and Dentistry, Riyadh Elm University, Riyadh, SAU; 2 College of Dentistry, King Saud bin Abdulaziz University for Health Sciences, Riyadh, SAU; 3 College of Dentistry, Princess Nourah Bint Abdul Rahman University, Riyadh, SAU; 4 Department of General Dentistry, College of Dentistry, King Khalid University, Abha, SAU

**Keywords:** radiographs, panoramic, symmetry, continuity, shape, position, mental foramen

## Abstract

Introduction: Many dental treatments need exact knowledge of the anatomical location of the mental foramen (MF). This retrospective research uses orthopantomograph (OPG) to assess the MF of orthodontic patients in Saudi Arabia and their position, symmetry, shape, and connectivity.

Materials and method: One hundred and eighty panoramic radiographs of patients having orthodontic treatment were analyzed for the location, form, symmetry, as well as bilateral preservation of the MF. The patients were of varied ages, genders, and degrees of malocclusion and were divided into three classes: Class I (60), Class II (60), and Class III (60). Chi-squared tests and other descriptive statistics were employed to examine the data for statistical significance.

Result: When looking at the right side of the mouth, the most frequent location for MF was position 3 (between the apexes of the first and second premolars; 50.6%), whereas on the left side, it was location 4 (near the apical of the second premolar; 47.2%). The most typical form of MF has an irregular shape. MF location on the left side and continuity types differed significantly across malocclusion groups (p<0.05).

Conclusion: OPGs of Saudi orthodontic patients demonstrated a significant variability of position and continuity of the MF across different classes of malocclusion. The third most frequent position was between the first and second premolars, while the shape of MF showed variation across the age of the patients. Hence, this precise comprehension of the anatomical and morphological diversity of the MF is of utmost significance for dental professionals.

## Introduction

The mental foramen (MF) is a significant anatomical landmark in the mandible. It is a bilateral, funnel-shaped aperture on the lower jaw's buccal surface. This anatomical characteristic most obviously reveals its inferior presentation in the interproximal region, in particular, in the hollow space between the roots of the lower jaw's premolar teeth. Branching off of the inferior alveolar nerve, the mental nerve (MN) passes through the MF [1].

The MN transmits sensory and motor impulses to teeth and gums in the mental region. Lower incisors, canines, and premolars are also included in this group [2]. The MF serves as a crucial reference point for diagnostic and clinical procedures. An accurate understanding of the particular location, form, and dimensions of a MF is crucial to achieving effective and uncomplicated dental operations, including surgical implant implantation, endodontic surgeries, and osteotomies within the specific area [3]. Not only dentists but also plastic surgeons and emergency medicine physicians require knowledge of the MF. These medical professionals perform a diverse range of surgical procedures, such as perioral (ear) surgery, orthognathic (jaw) surgery, lower lip and mandible laceration repair, and facial reconstruction [4]. When the neurovascular bundles of the MN are damaged, a serious complication known as paresthesia may occur after dental surgery [5]. The precise location of the MF may exhibit variability among different populations [6-8]. Various techniques, including cadaveric dissection, panoramic radiography, periapical radiographs, magnetic resonance imaging (MRI), computed tomography (CT), and cone beam computed tomography (CBCT), have been used to pinpoint the precise site of MF [9].

After conducting a comprehensive review of the literature, it was found that only a few studies have shown the prevalence of MF in a population from Saudi Arabia. These investigations were conducted in various parts of the nation, including the central, northern, and eastern regions, and used CBCT as the imaging technique [8,10,11]. The aforementioned research examined many anatomical characteristics of MF, including the placement of MF, the trajectory of the MN, and proximity to neighboring cortical plates.

Although CBCT is more precise in determining the location and form of the MF, it is not often used as a standard diagnostic method for orthodontic diagnosis. On the other hand, panoramic radiographs are often used in orthodontic diagnostics. Hence, panoramic radiographs may be used to ascertain the alignment and relative position of MF. The existing body of literature within standard radiological and anatomical textbooks presents notable differences of views concerning the precise positioning and morphology of the MF. The anatomical location of the MF exhibits variations across diverse ethnic populations worldwide.

The pretreatment evaluation of MF characteristics using panoramic radiographs of orthodontic patients is important to know about the MN course and its position about the adjacent teeth. This may help orthodontists avoid temporary MN paresthesia that may result secondary to orthodontic treatment. Based on the intended tooth movement and MN approximation, possible consequences of the orthodontic treatment to the patients can be explained beforehand. Moreover, MF is an important anatomical structure for planning a skeletal anchorage for orthodontic treatment. There were limited reports on the MF characteristics specifically among Saudi orthodontic patients. Hence, the objectives of this retrospective study were to examine the position and shape of the MF and its relevance to Saudi orthodontic patients using pretreatment panoramic radiographs.

## Materials and methods

A retrospective radiographic study of Saudi orthodontic patients undergoing (fixed or removable or surgical) treatment in various private university teaching hospitals in Riyadh City was carried out in Riyadh Elm University in Riyadh, Saudi Arabia. The ethical clearance for the study was obtained from the same institution's research and innovation center (approval number: SRP/2021/93/534/517). The pretreatment panoramic radiographs were included in this study. The panoramic radiograph taken at the diagnostic stage of orthodontic treatment was utilized in this study. None of the posttreatment panoramic radiographs were utilized in this study after a change in the position of the teeth after orthodontic movement.

Pretreatment orthopantomographs (OPGs) of patients between the ages of 18 and 45 who sought orthodontic treatment in three Riyadh Elm University hospitals were considered for the research. Patient X-ray exposure was not increased for this study; instead, the pretreatment panoramic images were retrieved from the patient's directory. This research examined a sample size of 180 high-quality radiographs of the MF (n=360), consisting of the left and right sides. Permanent dentition and the presence of teeth next to the MF on both sides of the jaw from the canine to the first molar were examined in the OPG to identify morphological features.

Cone-cut panoramic images that were blurry or otherwise unusable, patients with incomplete dentition, and those with obstructions to the MF region were not included in the study. Additional exclusions were the presence of radiographic abnormalities, mandibular fractures, and impacted, unerupted, or congenitally absent premolars. The Planmeca ProMax classic 2D machine (Planmeca, Helsinki, Finland) was used to capture the OPG pictures, and the Planmeca Romexis viewer software, version 5.1.0.R (Planmeca, Helsinki, Finland), was used to obtain all of the digital radiograph measurements related to the MF. The following parameters were used to capture OPG: at 70 kilovolts, for 16 seconds, and at 71 millisieverts per centimeter squared.

**Figure 1:**
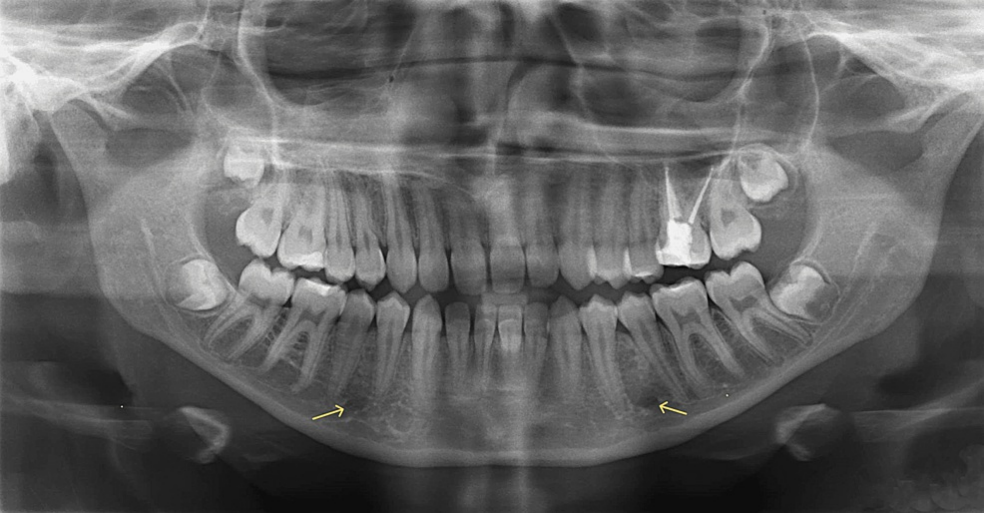
Panoramic radiograph showing MF on the third position and shape (yellow arrows) MF: mental foramen

**Figure 2:**
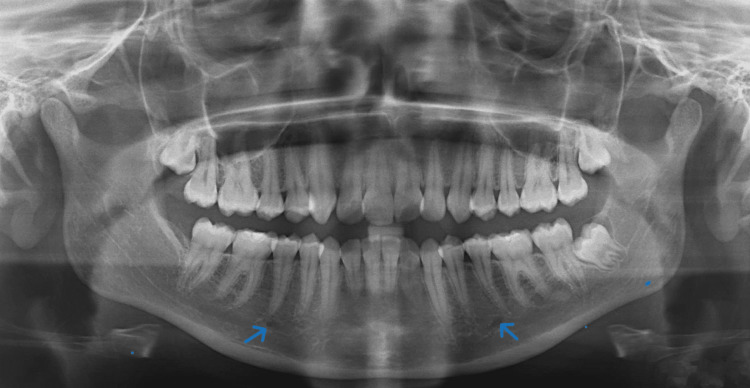
Panoramic radiograph showing MF on the fourth position and shape (blue arrows) MF: mental foramen

In this study, MF position, shape, and continuity on both sides of the jaws were noted as primary outcome variables. Secondary outcomes included the assessment of gender comparisons, evaluation of right and left differences, and study of bilateral symmetry. The MF's position relative to the other mandibular teeth was noted: first position: slightly in front of the apex of the first premolar; second position: at the very tip of the first premolar; third position: located around the same distance from either end as the first and second premolars; fourth position: the very tip of the second premolar; fifth position: between the very tips of the second premolar and the very tips of the first molar; and sixth position: up towards the top of the first molar. Figure 1 and Figure 2 show example OPGs using MF features.

If the two MF are in the same anteroposterior location concerning the teeth, the MF are said to be symmetrical. If the MF on each side of the mouth is not in the same anteroposterior location concerning the teeth, then the MF is asymmetrical. Yosue and Brooks's categorization system was used to analyze the bilateral continuity of the foramen: type I: the mental canal and the mandibular canal are the same; type II: the mandibular canal and foramen are anatomically different structures; type III: diffuse with a sharply defined foramen border; and type IV: cannot be classified. Multiple shapes, including oval, round, and irregular, were noted for MF. From the centers of two MF, the distance between them was calculated. Using dental imaging software, the distance between the two FM was calculated to be 1 millimeter.

The many types of malocclusion are cataloged and organized by Angle's classification. Class I occlusion describes the typical anteroposterior connection between the upper and lower first permanent molars, in which the mesiobuccal cusp of the top molar fills the groove of the lower molar. Class II malocclusion, also known as postnormal occlusion, is characterized by a distal connection between the mandibular and maxillary first molars (at least half of the distal cusp on each tooth). In Class III malocclusion, also called mesiocclusion, too much space exists between the mesiobuccal cusp of the upper first permanent molar and the mesiobuccal groove of the lower first permanent molar or between the lower first and second molars and the embrasure [12].

One investigator was tasked with the selection of OPGs by the inclusion and exclusion criteria to maintain consistency. The intra-examiner reliability showed a perfect agreement (kappa score=0.85). The visual assessment was employed to ascertain the characteristic appearance of MF. All the data obtained from the OPG were entered into the Excel sheet (Microsoft Corporation, Redmond, Washington, United States) and then transferred to the specialized statistical software.

Descriptive statistics were computed to analyze the frequency distribution and percentages of the categorical variables. The association of MF shape and position, the patient's age and gender, and the effects of malocclusion type and tooth crowding were analyzed using chi-squared and Fisher's exact tests. Spearman's test was applied to find the correlation between patient's age and MF positioning and shape. All tests were considered statistically significant if their p-values were less than 0.05. IBM SPSS Statistics for Windows, Version 25.0 (Released 2017; IBM Corp., Armonk, New York, United States) was used to analyze the data.

## Results

A total of 180 panoramic radiographs of orthodontic patients were carefully examined to assess the MF parameters. The study characteristics are described in Table 1.

**Table 1:** Characteristics of the study participants (N=180) n: number of participants; %: percentage

Characteristics		n	%
Age in years	≤20	83	46.1%
	>20	97	53.9%
Gender	Male	74	41.1%
	Female	106	58.9%
Angle's malocclusion classes	Class I	60	33.3%
	Class II	60	33.3%
	Class III	60	33.4%
Crowding	Yes	108	60.0%
	No	72	40.0%

A majority of the participants, comprising 97 individuals (53.9%), were aged older than 20 years, and the study included 106 female patients (58.9%). The orthodontic patients were evenly distributed among various classes of malocclusion. Furthermore, 108 orthodontic patients (60%) exhibited crowding of teeth.

Table 2 shows the distribution of the MF shape, position, and continuity between the right and left sides of the jaws.

**Table 2:** MF parameters between the right and left sides n: number of participants; %: percentage; MF: mental foramen p-value p<0.05 considered significant

MF variables		Right side		Left side		p-value
		n	%	n	%	
MF position	Position 1	1	0.6	4	2.2	0.402
	Position 2	15	8.3	17	9.4	
	Position 3	91	50.6	85	47.2	
	Position 4	58	32.2	65	36.1	
	Position 5	15	8.3	9	5.0	
MF shape	Round	79	43.9	75	41.7	0.633
	Oval	13	7.2	18	10.0	
	Irregular	88	48.9	87	48.3	
MF continuity	Type I	78	43.3	71	39.4	0.902
	Type II	81	45.0	86	47.8	
	Type III	13	7.2	14	7.8	
	Type IV	8	4.4	9	5.0	

Irregular MF shape, position 3, and type II continuity were predominantly observed on both sides. The analysis did not reveal any statistically significant associations between MF shape, position, and continuity with respect to sides (p>0.05).

Table 3 shows the association between malocclusion classes and MF position on the right and left sides.

**Table 3:** Association between malocclusion classes and MF position n: number of participants; %: percentage; MF: mental foramen p-value p<0.05 considered significant

Side	Position	Class I		Class II		Class III		p-value
		n	%	n	%	n	%	
Right	Position 1	0	0.0	0	0.0	1	1.7	0.506
	Position 2	7	11.7	3	5.0	5	8.3	
	Position 3	27	45.0	29	48.3	35	58.3	
	Position 4	22	36.7	21	35.0	15	25.0	
	Position 5	4	6.7	7	11.7	4	6.7	
Left	Position 1	2	3.3	1	1.7	1	1.7	0.021
	Position 2	7	11.7	5	8.3	5	8.3	
	Position 3	23	38.3	23	38.3	39	65.0	
	Position 4	27	45.0	26	43.3	12	20.0	
	Position 5	1	1.7	5	8.3	3	5.0	

On the right side of the jaws, there was no statistically significant association found between the position of MF and malocclusion classes (p=0.506). However, a noteworthy association was identified between malocclusion classes and MF on the left side (p=0.021).

Table 4 demonstrates the association between malocclusion classes and MF shape on the right and left sides.

**Table 4:** Association between malocclusion classes and MF shapes n: number of participants; %: percentage; MF: mental foramen p-value p<0.05 considered significant

Side	Shape	Class I		Class II		Class III		p-value
		n	%	n	%	n	%	
Right	Round	22	36.7	33	55.0	24	40.0	0.111
	Oval	3	5.0	6	10.0	4	6.7	
	Irregular	35	58.3	21	35.0	32	53.3	
Left	Round	19	31.7	31	51.7	25	41.7	0.253
	Oval	7	11.7	6	10.0	5	8.3	
	Irregular	34	56.7	23	38.3	30	50.0	

The shape of MF did not exhibit any statistically significant associations with malocclusion classes on either the right side (p=0.111) or the left side (p=0.253).

Table 5 exhibits the association between malocclusion classes and continuity of MF on the right and left sides.

**Table 5:** Association between malocclusion classes and continuity of MF n: number of participants; %: percentage; MF: mental foramen p-value p<0.05 considered significant

Side	Type	Class I		Class II		Class III		p-value
		n	%	n	%	n	%	
Right	Type I	44	73.3	19	31.7	15	25.0	<0.001
	Type II	8	13.3	36	60.0	37	61.7	
	Type III	0	0.0	5	8.3	8	13.3	
	Type IV	8	13.3	0	0.0	0	0.0	
Left	Type I	41	68.3	13	21.7	17	28.3	<0.001
	Type II	10	16.7	40	66.7	36	60.0	
	Type III	0	0.0	7	11.7	7	11.7	
	Type IV	9	15.0	0	0.0	0	0.0	

The continuity of MF displayed a significant association with malocclusion classes on both the right side (p<0.001) and the left side (p<0.001).

Table 6 shows the association of gender, age, and dental crowding with MF parameters.

**Table 6:** Association of gender, age, and crowding with MF parameters n: number of participants; %: percentage; MF: mental foramen p-value p<0.05 considered significant Position 1: slightly in front of the apex of the first premolar; position 2: at the very tip of the first premolar; position 3: located around the same distance from either end as the first and second premolars; position 4: the very tip of the second premolar; position 5: between the very tips of the second premolar and the very tips of the first molar; position 6: up towards the top of the first molar

Side	MF	Gender					Age (in years)					Dental crowding				
		Male		Female		p-value	≤20		>20		p-value	Yes		No		p-value
		n	%	n	%		n	%	n	%		n	%	n	%	
Right	Position 1	0	0.0	1	0.9	0.092	0	0.0	1	1.0	0.076	0	0.0	1	1.4	0.209
	Position 2	5	6.8	10	9.4		3	3.6	12	12.4		6	5.6	9	12.5	
	Position 3	33	44.6	58	54.7		49	59.0	42	43.3		58	53.7	33	45.8	
	Position 4	32	43.2	26	24.5		23	27.7	35	36.1		33	30.6	25	34.7	
	Position 5	4	5.4	11	10.4		8	9.6	7	7.2		11	10.2	4	5.6	
Left	Position 1	1	1.4	3	2.8	0.875	1	1.2	3	3.1	0.202	1	0.9	3	4.2	0.126
	Position 2	6	8.1	11	10.4		4	4.8	13	13.4		8	7.4	9	12.5	
	Position 3	35	47.3	50	47.2		44	53.0	41	42.3		57	52.8	28	38.9	
	Position 4	29	39.2	36	34.0		31	37.3	34	35.1		35	32.4	30	41.7	
	Position 5	3	4.1	6	5.7		3	3.6	6	6.2		7	6.5	2	2.8	
Symmetry	Symmetric	41	55.4	50	47.2	0.277	47	56.6	44	45.4	0.132	63	58.3	28	38.9	0.011
	Asymmetric	33	44.6	56	52.8		36	43.4	53	54.6		45	41.7	44	61.1	
MF shape (right side)	Round	34	45.9	45	42.5	0.06	43	51.8	36	37.1	0.007	52	48.1	27	37.5	0.367
	Oval	9	12.2	4	3.8		1	1.2	12	12.4		7	6.5	6	8.3	
	Irregular	31	41.9	57	53.8		39	47.0	49	50.5		49	45.4	39	54.2	
MF shape (left side)	Round	26	35.1	49	46.2	0.115	38	45.8	37	38.1	0.091	48	44.4	27	37.5	0.644
	Oval	11	14.9	7	6.6		4	4.8	14	14.4		10	9.3	8	11.1	
	Irregular	37	50.0	50	47.2		41	49.4	46	47.4		50	46.3	37	51.4	
MF continuity (right side)	Type I	27	36.5	51	48.1	0.261	27	32.5	51	52.6	0.016	34	31.5	44	61.1	0.001
	Type II	35	47.3	46	43.4		48	57.8	33	34.0		60	55.6	21	29.2	
	Type III	8	10.8	5	4.7		5	6.0	8	8.2		9	8.3	4	5.6	
	Type IV	4	5.4	4	3.8		3	3.6	5	5.2		5	4.6	3	4.2	
MF continuity (left side)	Type I	25	33.8	46	43.4	0.444	27	32.5	44	45.4	0.097	34	31.5	37	51.4	0.047
	Type II	37	50.0	49	46.2		48	57.8	38	39.2		60	55.6	26	36.1	
	Type III	8	10.8	6	5.7		5	6.0	9	9.3		9	8.3	5	6.9	
	Type IV	4	5.4	5	4.7		3	3.6	6	6.2		5	4.6	4	5.6	

No significant associations were observed between gender and any of the studied MF parameters on both sides (p>0.05). However, on the right side, the age of the study participants demonstrated a significant association with MF shape (p=0.007) and MF continuity (p=0.016). Additionally, dental crowding was significantly associated with MF symmetry (p=0.011), MF continuity on the right side (p=0.001), and MF continuity on the left side (p=0.047).

Spearman's test showed no significant correlations between patient's age and MF position and shapes on both the right and left sides in a studied sample as shown in Table 7. 

**Table 7:** Spearman's correlation test for MF position and shape on the right and left sides MF: mental foramen

Variables	Correlation coefficient	p-value
MF position (right side)	-0.015	0.845
MF position (left side)	-0.005	0.946
MF shape (right side)	0.033	0.660
MF shape (left side)	-0.002	0.979

## Discussion

It is reasonable to assume that there will be some degree of variation in anatomical structures. There have been reports of partial or total absence of the MF, as well as the presence of auxiliary routes and deviations from the normal oval form, in the published literature [13-15]. How far these MF variations affect the Saudi patients undergoing orthodontic treatment has not been thoroughly investigated about gender, age of treatment, and malocclusion types. Hence, this retrospective study examined the position, shape, continuity, and symmetry of the MF among Saudi orthodontic patients using OPG radiographs.

The majority of patients with MF were found to have it at position 3 (in the space between the tips of their first and second premolars) on both sides of their mouths. Position 4 (near the tip of their second premolar) came in second. According to earlier research, there is a significant regional variation in the incidence of MF in Saudi populations. 52.8% of people living in the eastern part of the Kingdom had their MF below their mandibular second premolar, according to research by Al-Mahalawy et al. [16]. Aldosimani et al. showed that the MF was located close to the mandibular second premolar in a statistically significant number (more than 68.1%) of participants from the central area of the Kingdom [17]. Furthermore, separate studies conducted by Mahabob et al. and Srivastava revealed that the location of MF below the second premolars was observed in the eastern and northern regions [10,11], respectively. These studies utilized CBCT images to disclose the MF, while in this study, OPG images were used to identify the position of the MF among the orthodontic patients. Furthermore, no significant difference between the position of the MF and gender was observed in this study. This finding is in line with previous studies conducted in Saudi Arabia [8,16]. Contrarily, a study conducted by Srivastava demonstrated a significant difference in the position of the MF between male and female genders. Possible explanations include gender-related differences in jaw size [11]. In the present study, the jaw size and tooth size were not used as reference points for locating the position of the MF. Contrarily, CBCT was used to evaluate the position of the MF in primary dentition and permanent dentition where differences in the tooth size and jaw size showed variable positions of the MF [11]. However, no statistically significant difference was noticed when comparing the position of MF across different age groups and the presence or absence of crowded teeth.

In this study, the position of MF differed significantly across different classes of malocclusion on the left side. This finding is contrary to the previously reported study in which no significant association between classes of malocclusion and the position of MF was observed among the Indian population [18]. In contrast to the spherical form often seen in Jordanian and Egyptian people, the irregular shape of the MF was more prevalent [19]. Furthermore, the oval form was more frequent among Sri Lankans than the round [20]. There was no significant difference observed in MF's shape across different malocclusion classes, gender, side of the jaw, and crowded dental arch. However, a significant difference in MF shape was observed about age. The younger age group ≤20 years showed a higher prevalence of round MF, while >20-year-olds demonstrated higher proportions of irregularly shaped MF.

Most of the MF showed a symmetrical distribution without any significant difference across gender and age. However, the presence of dental crowding showed significant differences in the symmetry of MF. This could be due to the growth of the jaw. The substantial correlation between malocclusion classes and MF continuity type across both arches is a striking result of our research. In addition, MF continuity differed significantly across the age and crowding status of arches. In this study, panoramic radiographs were used due to their ability to see a comprehensive region including both hard and soft tissues, enabling a more precise determination of the horizontal and vertical dimensions of the MF. The study findings indicate that there are variations in position, shape, symmetries, and continuity of MF. More specifically, malocclusion classes have shown a significant variation in the position of MF on the left side and the type of MF continuity. While MF shape and continuity demonstrated a significant variation between the age groups considered in this study, similarly, MF symmetry and type of continuity showed a significant difference between the presence and absence of dental crowding in the arch. Spearman's test indicated an insignificant negative correlation between the patient's age and MF position on the right and left sides. Similarly, a negative insignificant correlation between the patient's age and the left side of the MF shape and an insignificant positive correlation between the patient's age and the right side of the MF shape could be seen. This could be attributed to the restriction of age groups considered in this study. 

While acknowledging the limits of our limited sample size, our findings provide valuable insights into the Saudi population and contribute to the existing body of knowledge. The investigation did not include an examination of skeletal malocclusion or factors that could have impacted the study's outcomes. Furthermore, the OPG image was evaluated by dental interns and orthodontists with extensive expertise, as opposed to an oral radiologist. Instead of evaluating their own, they reached a collective decision. Inter-examiner calibration exercise was carried out using nine panoramic radiographs. Consensus was reached in cases where there was any disagreement of recording MF position. Future research should use these comprehensive elements to examine the MF's current state in a broader range of subpopulations and with a representative study sample.

## Conclusions

All standard radiological and anatomical textbooks contain conflicting statements regarding the location and shape of the MF. Moreover, the MF has been reported to vary in position in different ethnic groups. This particular study focused on pretreatment panoramic radiographic investigation of Saudi orthodontic patients to determine the variation in MF with regard to gender, relative position, and different classes of malocclusion.

Within the limitations of the study, it can be concluded that orthodontic patients demonstrated a significant variability of position and continuity of the MF across different classes of malocclusion. The most frequent location was position 3, between the tips of the first and second premolars. However, there was age-related heterogeneity in MF form. Similarly, MF continuity is affected by the age and presence of crowding. Hence, this precise comprehension of the anatomical and morphological diversity of the MF is of utmost significance for dental professionals. This information is important to avoid temporary MN paresthesia secondary to orthodontic treatment as reported in several studies. In addition, it has anatomical importance for skeletal anchorage.
